# Structure-Dependent Water Responsiveness of Protein Block Copolymers

**DOI:** 10.1021/acsabm.4c00045

**Published:** 2024-05-15

**Authors:** Jacob Kronenberg, Yeojin Jung, Jason Chen, Maria Jinu Kulapurathazhe, Dustin Britton, Seungri Kim, Xi Chen, Raymond S. Tu, Jin Kim Montclare

**Affiliations:** † Department of Chemical and Biomolecular Engineering, New York University Tandon School of Engineering, Brooklyn, New York 11201, United States; ‡ Advanced Science Research Center (ASRC) at the Graduate Center, City University of New York, New York, New York 10031, United States; § Department of Chemical Engineering, City College of New York, New York, New York 10031, United States; ∥ PhD Programs in Chemistry and Physics at the Graduate Center, City University of New York, New York, New York 10016, United States; ⊥ Department of Chemistry, New York University, New York, New York 10031, United States; # Department of Biomaterials, New York University College of Dentistry, New York, New York 10010, United States; ∇ Department of Radiology, New York University Grossman School of Medicine, New York, New York 10016, United States; ○ Department of Biomedical Engineering, New York University Tandon School of Engineering, Brooklyn, New York 11203, United States

**Keywords:** protein engineering, water-responsive materials, biomaterials, protein films, block copolymers

## Abstract

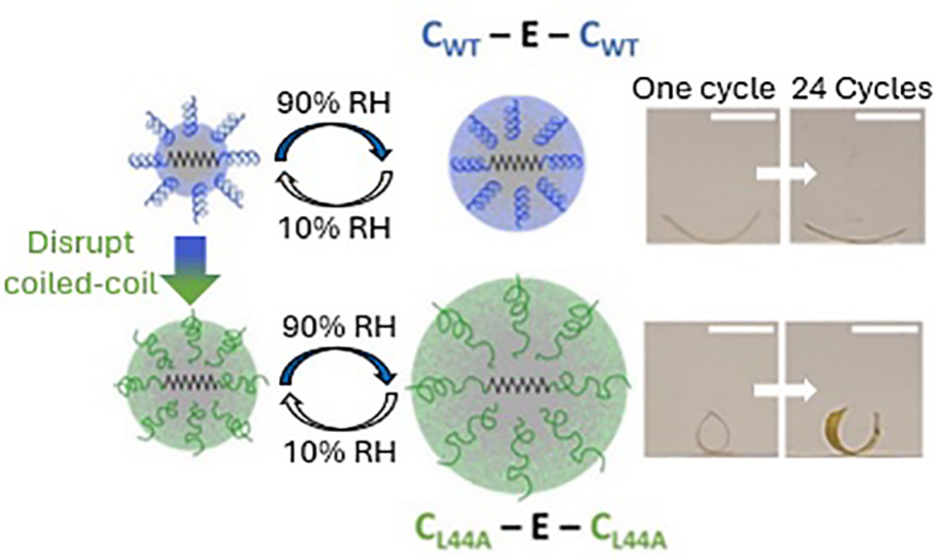

Biological water-responsive (WR) materials are abundant in nature, and they are used as mechanical actuators for seed dispersal by many plants such as wheat awns and pinecones. WR biomaterials are of interest for applications as high-energy actuators, which can be useful in soft robotics or for capturing energy from natural water evaporation. Recent work on WR silk proteins has shown that β-sheet nanocrystalline domains with high stiffness correlate with the high WR actuation energy density, but the fundamental mechanisms to drive water responsiveness in proteins remain poorly understood. Here, we design, synthesize, and study protein block copolymers consisting of two α-helical domains derived from cartilage oligomeric matrix protein coiled-coil (C) flanking an elastin-like peptide domain (E), namely, CEC. We use these protein materials to create WR actuators with energy densities that outperform mammalian muscle. To elucidate the effect of structure on WR actuation, CEC was compared to a variant, CEC_L44A_, in which a point mutation disrupts the α-helical structure of the C domain. Surprisingly, CEC_L44A_ outperformed CEC, showing higher energy density and less susceptibility to degradation after repeated cycling. We show that CEC_L44A_ exhibits a higher degree of intermolecular interactions and is stiffer than CEC at high relative humidity (RH), allowing for less energy dissipation during water responsiveness. These results suggest that strong intermolecular interactions and the resulting, relatively steady protein structure are important for water responsiveness.

## Introduction

Water-responsive (WR) biological materials are abundant in nature, with many plants relying on WR actuation to carry out important functions such as seed dispersal.^1^ Given the high energy density of previously reported WR biomaterials,^2^ there is increasing interest in the development of WR materials and using them as high-energy actuators for engineering applications.^3,4^ In these WR materials developed by nature, microstructures with different configurations control a variety of actuation modes including linear movement such as by spider silk, bending such as by pinecones or wheat awns, and twisting such as by *Bauhinia* seedpods.^2,5,6^ These microstructures typically show distinct hydrophobic/hydrophilic domains as well as soft/stiff domains. The soft domains take up water, expand, and push apart stiff domains, converting the chemical potential of water into mechanical energy.

The importance of these microstructures has been demonstrated in artificial WR actuators as well. In WR protein materials derived from *Bombyx mori* silk, energy density correlates with the presence of rigid, highly crystalline β-sheet domains.^7^ Water responsiveness can also be improved through the construction of composite materials with rigid inorganic nanoparticles enmeshed in a protein material, underscoring the importance of the presence of stiff domains to facilitate high WR energy densities.^8^

Protein block copolymers (PBCs) are highly controllable materials made with proteins consisting of multiple domains fused into a single protein chain.^9^ PBCs allow us to precisely test this ratio of stiff and soft domains in order to control water responsiveness. PBCs allow for highly tunable mechanical properties and response to a wide variety of stimuli through selection of blocks from a diverse toolkit of protein motifs, such as elastin-like peptides, silk-like peptides, and coiled-coils.^10^

Here, we design and develop protein block copolymers for WR actuators. Since previous work shows the importance of rigid domains interspersed in soft domains, we select a copolymer bearing a soft, unstructured domain with a rigid, highly structured domain. For the unstructured domain, we selected an elastin-like peptide (E) domain, a widely used peptide domain derived from a tandem repeat motif in the protein elastin. Elastin-like peptides are intrinsically disordered but exhibit a tunable temperature response linked to hydrophobic collapse at high temperatures.^11,12^ For the structured domain, we selected the cartilage oligomeric matrix protein coiled-coil (C) domain, an α-helical domain that self-assembles into coiled-coil pentamers.^13^

**Figure 1:**
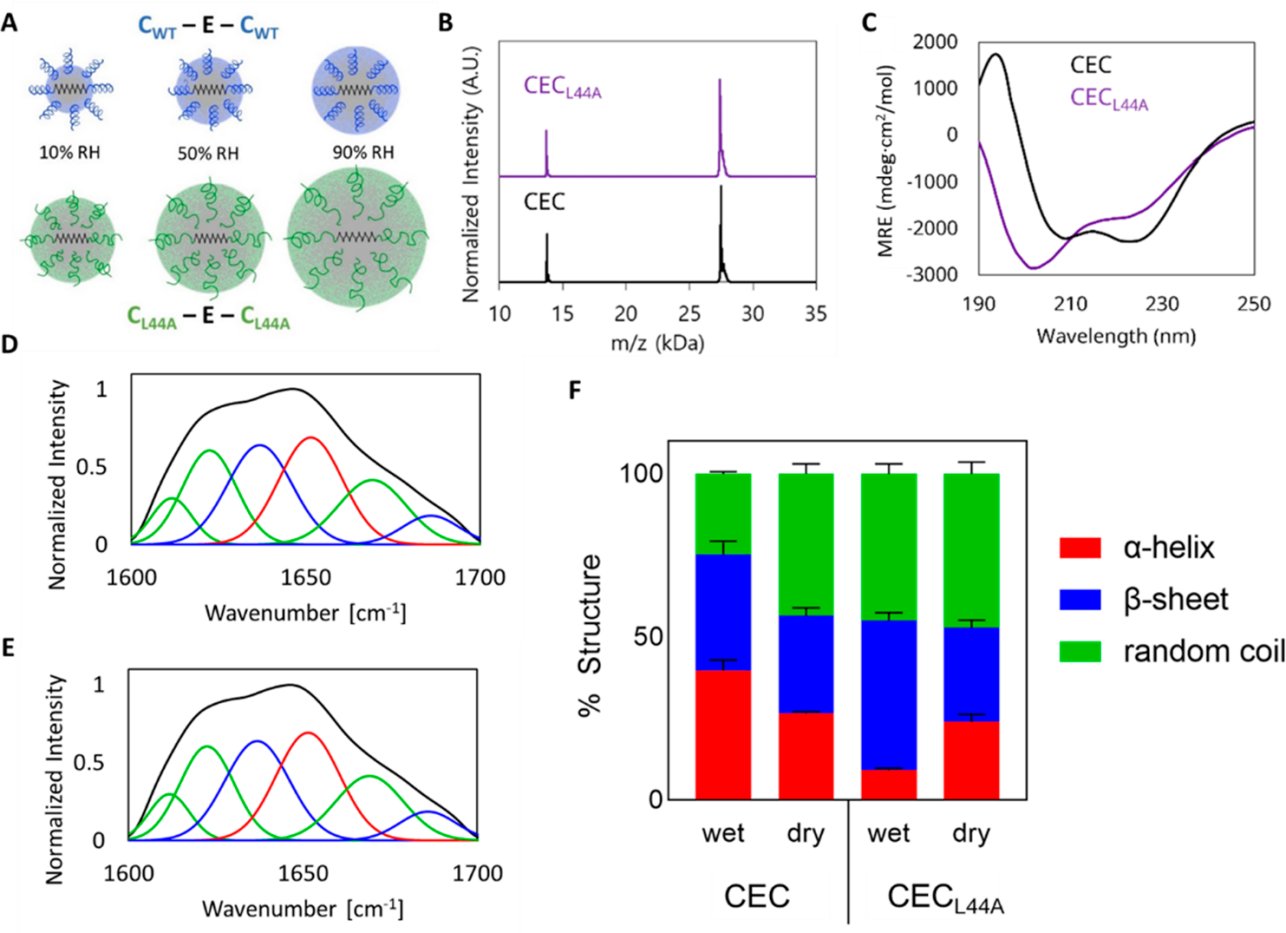
(A) Hypothetical schematic showing a small amount of swelling in CEC and a larger amount of swelling in CEC_L44A_, which leads to greater water response. (B) A representative MALDI MS spectrum confirming showing the CEC^+1^/CEC_L44A_^+1^ peak at 27.4 kDa and the CEC^+2^/CEC_L44A_^+2^ peak at 13.7 kDa. (C) CD spectra showing a difference in structure between highly α-helical CEC and disordered CEC_L44A_. (D) Representative ATR-FTIR spectra of dried CEC films; α-helical content shown in red, β-sheet content shown in blue, random coil content shown in green. (E) Representative ATR-FTIR spectra of dried CEC_L44A_ films. (F) Percent contributions to secondary structure of CEC and CEC_L44A_ as estimated by CD (wet) and ATR-FTIR spectroscopy (dry) revealing the change in structured contents (red+blue) between wet and dry conditions. Error bars indicates standard deviations calculated from three independent measurements.

We use CEC to develop WR bilayer actuators. To study the importance of secondary structure on water responsiveness, we also develop WR actuators using CEC_L44A_, a construct in which the C domains are disrupted by mutating the leucine in position 44 to alanine that results in loss of structure.^13,14^ While both CEC and CEC_L44A_ show water response, the energy density of CEC_L44A_ is 751.7 ± 66.9 kJ/m^3^, which is over seven times that of CEC with 100.3 ± 9.1 kJ/m^3^. Both materials lose energy density after repeated cycling, but CEC_L44A_ is more robust, losing less than a third as much energy density as CEC. We hypothesize that this difference is the result of less chain rigidity and subsequent chain flexibility of CEC_L44A_ (Figure 1A). At high relative humidity (RH), CEC_L44A_ retains a higher Young’s modulus than CEC, suggesting that its increased energy density comes from reduced energy dissipation. We demonstrate the role of secondary structure and water uptake in the two BCPs, revealing how self-assemblies that include unstructured domains can promote high WR energy densities.

## Materials and Methods

### Materials

Urea, imidazole, sodium phosphate dibasic, sodium phosphate monobasic, magnesium sulfate, calcium chloride, ammonium chloride, sodium chloride, potassium phosphate monobasic, ampicillin, chloramphenicol, thiamine, methanol, acetic acid, hydrochloric acid, glucose, glycerol and lactose were obtained from Fisher Scientific. HisPur Ni-NTA resin, sinapinic acid, ProteoMass Matrix-assisted laser desorption/ionization peptide standards and SnakeSkin 10 kDa MWCO dialysis tubing were obtained from Thermo Scientific. 30% acrylamide solution and gel electrophoresis equipment were obtained from BioRad. Polyimide film was obtained from CAPLINQ and cut to 1 cm strips before use.

### Protein Block Copolymer Synthesis and Purification

Protein block copolymers were expressed and purified as previously described.^14^ Briefly, AFIQ *E. coli* cells were transformed with pQE-30 vectors containing genes coding for CEC and CEC_L44A_ and grown on tryptic soy agar with ampicillin (Amp) and chloramphenicol (Cam) to select for successful transformants.^15^ Colonies were picked and grown overnight at 37 °C and 350 rpm in 10 mL complete M9 media (0.5 M Na_2_HPO_4_, 0.22 M KH_2_PO_4_, 0.08 M NaCl, and 0.18 M NH_4_Cl) supplemented with 10 mg/L of each of the 20 canonical amino acids, 1 mM MgSO_4_, 0.1 mM CaCl_2_, 0.2% w/v glucose, 0.2% v/v trace metal solution, 200 μg/mL Amp, 34 μg/mL Cam, and 35 μg/mL thiamine. The following day, expression cultures consisting of 400 mL complete M9, similarly supplemented, but with the glucose replaced with 50/52 (0.5% w/v glycerol, 0.05% w/v glucose, and 0.2% w/v lactose) for autoinduction.^16^ Cultures were incubated at 37 °C at 350 rpm for 8 h, then harvested by centrifugation at 4,000 rpm for 10 min at 4 °C in an Avanti J15 centrifuge. Samples were examined with sodium dodecyl sulfate polyacrylamide gel electrophoresis (SDS-PAGE) to confirm the presence of the protein of interest in the postinduction culture medium (Figure S1A**)**.

Cells were resuspended in buffer A (50 mM Na_2_HPO_4_, 6 M urea, 20 mM imidazole, pH 8) and lysed by sonication for 2.5 min at 60% amplitude with a QSonica Q500 probe sonicator. Lysate was clarified by centrifugation at 10,000 rpm for 45 min at 4 °C using an Avanti J15 centrifuge, then incubated overnight with Ni-NTA resin beads. The beads were washed with buffer A to remove unbound protein, then the protein of interest was eluted using increasing concentrations of buffer B (50 mM Na_2_HPO_4_, 6 M urea, 500 mM imidazole, pH 8). Fractions determined by SDS-PAGE to be pure (Figure S1B) were dialyzed first against 50 mM Na_2_HPO_4_ with decreasing concentrations of urea (4 M, 2 M, 1 M, 0.5 M, 0.25 M) to allow the protein to refold without aggregation, then dialyzed against deionized water to remove all salt. Dialyzed samples were flash-frozen and lyophilized to obtain pure protein samples as a powder.

To confirm the purity of protein samples, Matrix-assisted laser desorption/ionization time-of-flight mass spectrometry (MALDI-ToF MS) was performed on dialyzed protein samples. Aqueous samples were mixed 1:1 and 1:2 with a saturated solution of sinapinic acid (SA) in 50% v/v acetone and 0.1% trifluoroacetic acid (TFA). A ground steel MALDI target plate from Bruker was spotted with 1 μL of sample and dried under vacuum. Samples were flown on a Bruker ultrafleXtreme MALDI-ToF mass spectrometer in reflect1 Fr mode at 80% laser intensity. The instrument was calibrated using apomyoglobin (16.9 kDa), aldolase (39.2 kDa) and bovine serum albumin (66.4 kDa) spotted with an identical matrix.

### Circular Dichroism Spectroscopy

Circular dichroism (CD) spectra were measured using a JASCO J-815 Spectropolarimeter using Spectra Manager software. A quartz cuvette (Hellma Analytics) with a 1 mm path length was loaded with 400 μL protein solution. All measurements were taken with protein samples diluted to 10 μM in 50 mM sodium phosphate buffer at pH 8. Spectra were collected at 5 °C increments from 25 to 85 °C at a rate of 1 nm/s and the ellipticity at 222 nm (θ_222_) was measured at 1 °C increments. Spectra were smoothed using a Savitzky-Golay filter^17^ and converted to mean residual ellipticity.^13^ Secondary structure was estimated by deconvolution with BeStSel.^18^

### Infrared Spectroscopy

Attenuated total reflectance Fourier transform infrared (ATR-FTIR) spectra were collected using a Nicolet iS50 Fourier transform infrared spectrometer (Thermo Scientific) with a DTGS-KBr accessory. The spectrometer was blanked with 5 μL of deionized water then 5 μL of protein sample in deionized water was spotted onto the stage and dried. Spectra were taken as the average of 128 accumulations from 4000 to 400 cm^–1^ at room temperature with a penetration depth of approximately 1 μm. The amide I band (1600–1700 cm^–1^) was studied to determine protein secondary structure. PeakFit (Systat Software, Inc.) was used to deconvolute the spectra using a Gaussian model, and deconvoluted peaks were assigned to protein structure types and used to approximately quantify the secondary structure content as previously reported.^7,19,20^

### Water Responsiveness Characterization

Lyophilized protein samples were resuspended in deionized water to a concentration of 10 mg/mL. A 25 μm thick polyimide substrate was cut into 3 mm width by 6 mm length strips and treated with a NanoClean plasma oxidizer (Fischione) to improve surface adhesion. Protein samples with a thickness of 8 μm were cast onto the polyimide substrate. Bilayer strips were placed in a RH control chamber and their curvature was measured at 10% and 90% RH. To determine the reversibility of the actuation, measurements were taken over the course of one cycle as well as after 24 cycles. ImageJ software was used to quantify their curvature, and energy density was calculated from the energy of bending of the strips and protein volume as previously described.^3,7^
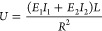
1

Energy of bending was calculated by eq 1, where *R* is the radius of curved strip, *I*_1_ and *I*_2_ are the area moment of inertia, and *E*_1_ and *E*_2_ (2.5 GPa) are Young’s moduli of the protein and the polyimide substrate, respectively. *E*_1_ is measured by AFM (Figure 5B).

### Dynamic Water Vapor Sorption

The water sorption isotherms of CEC and CEC_L44A_ were measured using a dynamic vapor sorption (DVS) instrument (Intrinsic Plus, Surface Measurement Systems) as previously described.^7^ Briefly, 15 strip samples were placed in the quartz ultrasensitive microbalance pans under a RH-controlled atmosphere, and changes in the samples’ masses were recorded at 25 °C over the course of three RH cycles. Each RH cycle consisted of 10% RH steps from 10% RH up to 90% RH and back down to 10% RH. The RH was held at each step until the change in mass per unit time was less than 0.02%/min for at least 30 min and the maximum time was fixed to be 60 min. To determine the water sorption behavior of the lyophilized protein exclusively, a separate DVS test was conducted using only polyimide substrates without depositing the protein. The mass change of the polyimide films was subtracted from the total mass change of the strips. This subtraction allowed us to specifically obtain the water sorption isotherms related to the 2 mg of lyophilized protein. Mass percent vapor sorption was calculated according to eq 2, where *m* is the total mass of the sample at a given % RH and *m*_*10%RH*_ is the mass of the sample at 10% RH.
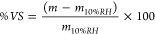
2

### Young’s Modulus Estimation

Atomic force microscopy (AFM, Multimode 8, Bruker) nanoindentation was performed on cast protein thin layers to determine their Young’s modulus as previously described.^7^ A 10 mg/mL solution of diluted proteins was deposited onto a silicon wafer, resulting in the formation of a layer with a thickness of approximately 6–8 μm. The Young’s modulus of the proteins was measured using tapping mode under ambient conditions (approximately 25 °C and 30% RH). An AFM tip with a radius of 8 nm (NCHV, Bruker) was employed. The deflection sensitivity of the cantilever was determined as 47.2 nm/V through a ramp curve measurement on a silicon wafer, while the spring constant was calibrated as 2.92 N/m using the thermal tune mode. The obtained force–separation curves were analyzed using Nanoscope analysis software, with the retract line selected for baseline fitting. The force range of 5–75% was fitted using the Hertzian (Spherical) model to estimate the Young’s modulus.

### Electron Microscopy

The surface morphologies of the CEC and CEC_L44A_ strips were characterized using a Zeiss MERLIN scanning electron microscope (SEM). Strips were dried and sputter coated with gold at a thickness of 3.5 nm, then imaged in scanning mode at a potential of 5 kV at magnifications ranging from 1kx to 30kx. Strips of both materials were characterized after having undergone a single wet/dry cycle or 24 cycles. Bare polyimide, which is used as the passive layer in the bilayer actuators, was also imaged as a control (Figure S4). Surface porosity was estimated using ImageJ software.

Transmission electron microscopy (TEM) was used to characterize the self-assembly of CEC and CEC_L44A_. To mimic WR actuator casting conditions, protein samples at 10 mg/mL in deionized water were spotted onto Formvar/carbon-coated copper grids and dried for 1 min at ambient conditions. Samples were stained with 3 μL 1% uranyl acetate for 1 min, then blotted and dried at ambient conditions. Samples were imaged at 130 kV on a JEOL1400 Flash TEM at NYU Langone’s Microscopy Laboratory. Particle size was estimated using ImageJ software as an average of 15 representative particles.

## Results and Discussion

### Protein Block Copolymer Expression and Structure

Both CEC and CEC_L44A_ were expressed and purified as done previously.^14^ The presence of the proteins of interest was confirmed using SDS-PAGE after expression (Figure S1A) and purification (Figure S1B). MALDI-ToF MS confirmed purity with masses of 27.4 kDa for both proteins (Figure 1B). The secondary structure of CEC and CEC_L44A_ was assessed by circular dichroism (CD) at 25 °C (Figure 1C). As expected, CEC showed minima of −1,450 ± 230 deg·cm^2^/mol at 208 nm and −1,730 ± 34 deg·cm^2^/mol at 222 nm, consistent with previously reported data.^14^ In contrast, CEC_L44A_ demonstrated a minimum of −3,590 ± 240 deg·cm^2^/mol at 204 nm and lost signal at 222 nm, confirming a loss of secondary structure. Deconvolution of CD spectra using the Beta Structure Selection (BeStSel)^18^ tool revealed that the L44A mutation reduced α-helical content from 39.7 ± 1.9% to 9.1 ± 1% and increases β-sheet content from 17.4 ± 2.3% to 28.3 ± 1.4% (Table S1).

In addition to CD for solution-phase structure, ATR-FTIR was conducted on dried films of the two block copolymers (Figure 1D,E). ATR-FTIR amide I bands were deconvoluted into five subpeaks corresponding to α-helices, random coils, and β-strands.^19^ Structural characterization by FTIR demonstrates that the films result in a very different secondary structure distribution to the solvated BCPs. In the film, CEC and CEC_L44A_ formed α-helices, at 26.5 ± 0.6% and 24.0 ± 2.3%, respectively. Additionally, CEC and CEC_L44A_ showed a β-sheet content of 30.0 ± 2.4% and 28.8 ± 2.3%, respectively. The results show that CEC and CEC_L44A_ transform into a similar secondary structure as measured by FTIR. The difference between the CD measurements and FTIR measurements indicated that a higher structured content in CEC compared to CEC_L44A_ on both wet and dried states, where wildtype CEC possesses more α-helical content and less β-sheet content than CEC_L44A_ under wet conditions (Figure 1F). CEC_L44A_, in contrast to CEC, revealed a greater exchange within the structured content between wet and dried conditions, where the difference in α-helical content is 14.9% for CEC_L44A_ and 13.2% for CEC, while the difference in β-sheet content is 17.1% for CEC_L44A_ and 5.6% for CEC.

### Effect of Structure on WR Actuation and Reversibility

**Figure 2:**
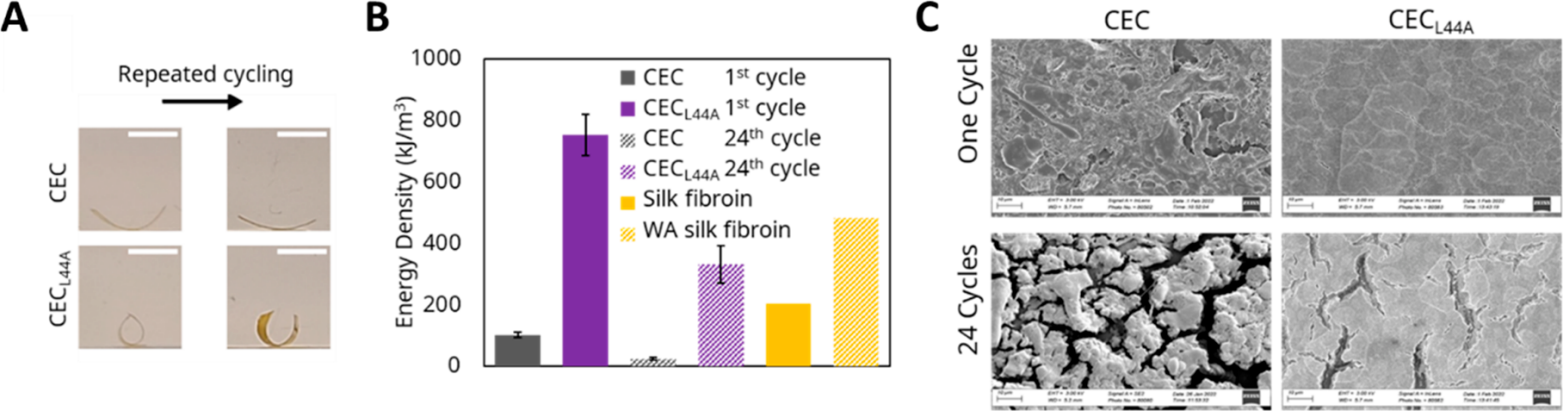
(A) Sample images of the bilayer actuators at 10% RH after one cycle and after 24 cycles, showing reduced curvature after repeated cycles, reflecting loss of energy density. Scale baris 1 cm. (B) WR energy densities of CEC and CEC_L44A_ on their first cycle and 24th cycle, compared with previously reported WR material *Bombyx mori* silk fibroin before and after water vapor annealing (WA).^5^ (C) SEM micrographs showing representative surfaces of CEC and CEC_L44A_ films after one RH cycle and after 24 RH cycles, showing moderate degradation of CEC_L44A_ and significant degradation of CEC. Scale bar is 10 μm.

Both CEC and CEC_L44A_ demonstrated WR actuation in response to changes between 90% and 10% RH (1 in Figure 2A). CEC had an energy density of 100.3 ± 9.1 kJ/m^3^, but CEC_L44A_ outperformed it with an energy density of 751.7 ± 66.9 kJ/m^3^ (Figure 2B). Remarkably, CEC_L44A_ exceeded the energy density of natural actuators such as mammalian muscle, as well as spider silk—one of the strongest natural WR protein materials known, with a WR energy density of 500 kJ/m^3^.^21^ It significantly outperforms mammalian muscle, which has an energy density of 8 kJ/m^3^.^7^

CEC and CEC_L44A_ were subjected to 24 WR cycles to study the reversibility and robustness of their actuation. Both proteins exhibited a drop in energy density after repeated cycling (Figure 2B). However, this drop was significantly more pronounced for CEC, whose energy density diminished by nearly 80% to 22.7 ± 4.4 kJ/m^3^, as opposed to CEC_L44A_ whose energy density was only reduced by about half to 330.3 ± 60.0 kJ/m^3^. CEC_L44A_ shows better reversibility than CEC. Differences in SEM micrographs taken after a single cycle and after 24 cycles are consistent with the degradation in WR actuation energy density (Figure 2C). After a single cycle, the surfaces of both were largely intact. After 24 cycles, CEC_L44A_ had developed several visible fractures, whereas CEC was significantly damaged, almost to the point of being discontinuous. ATR-FTIR was used to assess structural changes after cycling CEC and CEC_L44A_ films for 24 cycles where both CEC_L44A_ and CEC showed negligible differences in β-sheet conformation compared to precycled films (Figure S2, Table S1**)**. Notably, both CEC and CEC_L44A_ films possessed a large increase in α-helical conformation after cycling with 7.2% and 11.1%, respectively, and decrease in random coil conformation of −4.2% and −9.9%, respectively. The observed molecular assembly of the block copolymer domains over time, particular of the coiled-coil domain, supported by previous work, demonstrates that increased aggregation and decreased material strength is the result of cycling.^22^ While both CEC and CEC_L44A_ suggest an increase in α-helical conformation after cycling, the C_L44A_ domain of CEC_L44A_ lacks strong coiled-coil self-assembly resulting in a lack of intercoil–coil interactions. Thus, the CEC_L44A_ film exhibits decreased fatigue due to a higher degree of intermolecular interactions, whereas CEC shows increased coiled-coil formation and intercoiled-coil interactions.

### Effect of Structure on Water Adsorption

DVS was used to measure the amount of water uptake by the materials during hydration and dehydration processes. When working with bulk lyophilized protein, CEC adsorbed 10% more vapor per unit mass between 10% RH and 90% RH than CEC_L44A_ (Figure S3). Both proteins exhibited hysteresis when subjected to multiple cycles.

**Figure 3:**
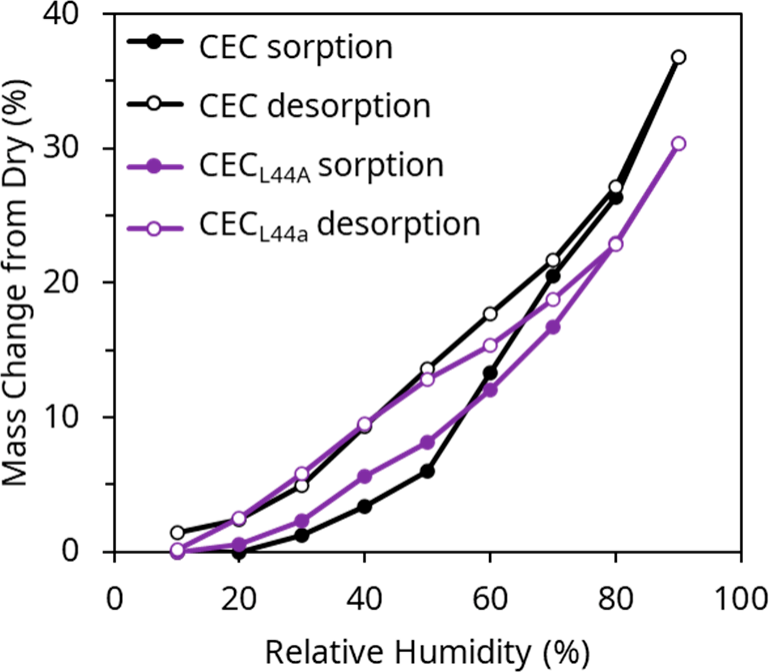
DVS water sorption isotherms showing that CEC film takes up slightly more water than CEC_L44A_ film and that both show minimal hysteresis during desorption.

Analysis of SEM micrographs of the cast proteins suggested a difference in macrostructure between CEC and CEC_L44A_ thin films that could affect water adsorption behaviors in ways that are not reflected in bulk studies (Figure S3). Therefore, we directly took DVS measurements of CEC and CEC_L44A_ (Figure 3) cast films. Overall, we observed the same trends for cast protein as bulk protein, indicating that macrostructure did not significantly affect water adsorption. Interestingly, CEC showed a slightly greater maximum water adsorption than CEC_L44A_, despite CEC exhibiting a lower WR actuation energy density than CEC_L44A_. Both proteins displayed similar hysteresis over time. One possible explanation for this is that CEC self-assemblies bury the hydrophobic residues inside of a coiled-coil pore, but the L44A mutation inhibits this assembly, so that hydrophobic residues are more surface-exposed. The decrease in favorable interactions could lead to less water adsorption by CEC_L44A_ at the same RH.

### Macroscopic Assembly and Surface Characterization

**Figure 4:**
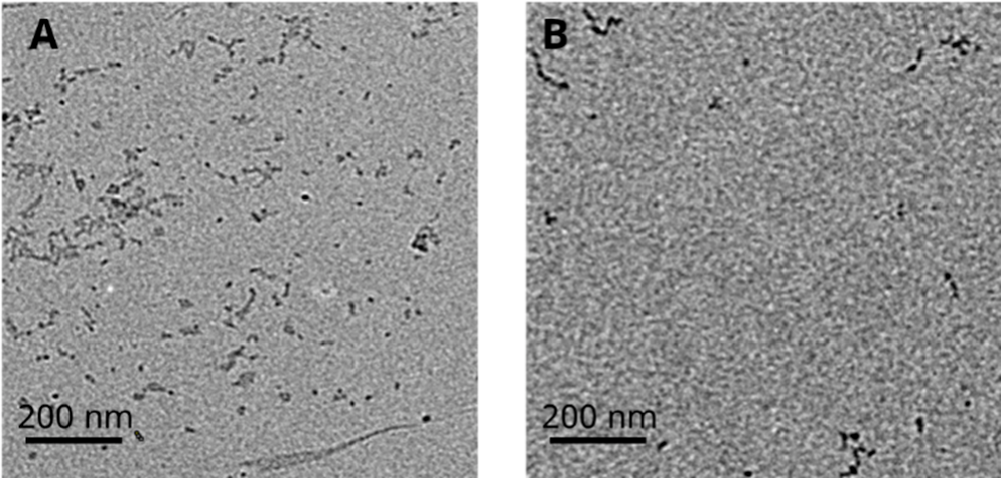
TEM micrographs showing particulate assemblies by CEC (A) and CEC_L44A_ (B).

CEC and CEC_L44A_ assemblies were imaged with TEM (Figure 4). Both proteins formed spherical particles when spotted in deionized water, similar to those previously reported in 10 mM phosphate buffer at pH 8.^14^ This confirms the self-assembly of the proteins under the conditions used to deposit BCPs into active “strip” layers. The particle diameter of CEC was 13.6 ± 2.5 nm and the particle diameter of CEC_L44A_ was 13.8 ± 1.7 nm. There is no difference between particle sizes of the two proteins after being dried for TEM, but it is possible that the sizes of their particles are different when hydrated.

**Figure 5:**
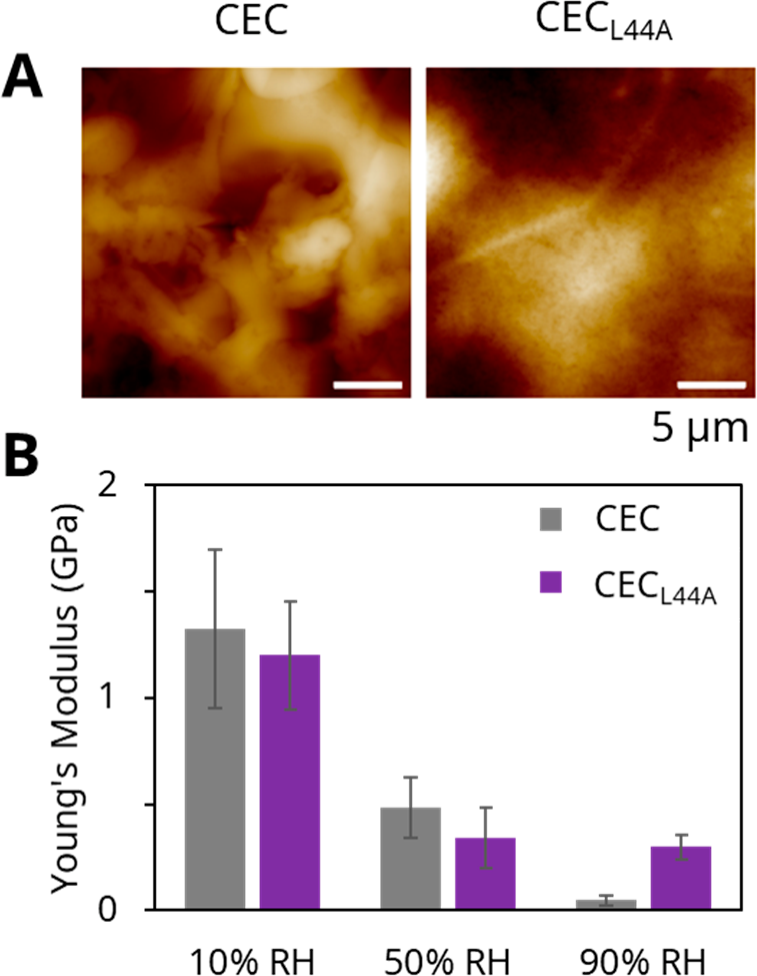
(A) AFM images of CEC and CEC_L44A_ cast films. (B) Young’s moduli of CEC and CEC_L44A_ measured by AFM at 10%, 50%, and 90% RH, showing greater maintenance of stiffness by CEC_L44A_ at high RH.

AFM was used to further assess the surface topology and to quantify mechanical properties. AFM provided some insight into differences between the two protein block copolymer surfaces. CEC exhibited a slightly rougher surface than CEC_L44A_ (Figure 5A). The Young’s modulus at 10% RH of CEC_L44A_ was 1.20 ± 0.26 GPa, a negligible difference compared to that of CEC, which was 1.32 ± 0.37 GPa (Figure 5B, Figure S5). Interestingly, at 90% RH, the Young’s modulus of CEC_L44A_ is 0.30 ± 0.06 GPa, higher than that of CEC, which drops to 0.05 ± 0.02 GPa. The relatively high stiffness of CEC_L44A_ at high RH could correlate with enhanced β-sheet content compared to CEC under wet conditions (Figure 1C, 1F, Table S1). Furthermore, the capability of CEC_L44A_ to maintain stiffness from 50% RH to 90% RH also suggest that some of its improved energy density may be the result of increased β-sheet domains, which agrees with our previous observation that β-sheet content correlates with WR energy density.^7^

### Correlation between Macrostructure, Domain Properties, and Water Responsiveness

The fact that CEC_L44A_ outperforms CEC suggests that greater resistance to change in structured content when drying confers increased water responsiveness. Since structural characterization shows greater β-sheet content in wet CEC_L44A_ and the previously reported work has shown that β-sheet content correlates with WR energy density,^7^ it is possible that the fold of C domain is irrelevant, rather the folding and refolding of the β-sheet composition of the overall protein including the C_L44A_ and E domains is important.

Since CEC contains two rigid C domains and CEC_L44A_ does not contain any rigid domains, this difference is likely explained by chain mobility in the C-block of the assembly. The greater chain mobility of CEC_L44A_ could mean that for the similar amount of water adsorption, it will undergo a greater change in volume.

The degree of the intermolecular interactions of the BCPs also appears to be important to WR energy density and reversibility. DVS experiments show that a relatively constant amount of water is adsorbed and desorbed over repeated cycles, but the similar amount of water results in weaker actuation. If the active layer fractures significantly, then any expansion resulting from water adsorption will result in discontinuous domains expanding into the cracks between them rather than in the bulk expansion necessary for actuation. CEC shows discontinuities that extend across the entire field of view whereas CEC_L44A_ shows only some discrete fractures (Figure 2C), which correlates with their greater and lesser respective loss of energy density. Lastly, the C_L44A_ domain is softer than the C domain, and may be less prone to aggregation due to less ordered coiled-coils,^22^ which allows for enhanced intermolecular interactions. Because of this, CEC_L44A_ is less likely to deform under repeated strain, which results in greater resilience after repeated cycling.

## Conclusions

The coiled-coil forming triblock protein copolymers, CEC, and its unstructured analogue CEC_L44A_ were cast into films, which served as the active layers of WR actuators. They both showed water response, but the energy density of CEC_L44A_ was significantly higher than that of CEC. The increased change in β-sheet content in wet and dry secondary structure measurements of CEC_L44A_ compared to CEC was consistent with in the higher WR energy density of CEC_L44A_, suggesting that protein structure is important to water response. CEC_L44A_ was also measurably stiffer at high RH than CEC and exhibited much less degradation over repeated cycling. Taken together, these results suggest that the increased energy density of CEC_L44A_ stems from increased β-sheet content at higher RH resulting in resistance to energy dissipation. Additionally, the higher relative random coil conformation of CEC_L44A_ at low and high RH may also facilitate the WR energy transfer from nanoscale to macroscale as a result of its higher intermolecular interactions. Future work will focus on mechanistic elucidation of water response by CEC_L44A_ and the use of new types of blocks in these block copolymers to further develop our understanding of the parameters of WR protein materials.

## Supporting Information Available

Description and SDS-PAGE images for CEC and CEC_L44A_ expression and CEC_L44A_ purification. Description and table for secondary structure content percentage using CD and ATR-FTIR for CEC and CEC_L44A_. Description of surface characterization and scanning electron micrograph images for CEC, CEC_L44A_, and control substrate at 10 kx and 30 kx resolution. Figure for DVS water sorption isotherms for lyophilized CEC and CEC_L44A_ powder. Representative figures for force–separation curves for AFM Young’s moduli of CEC and CEC_L44A_. (PDF)

## References

[ref1] Elbaum R.; Zaltzman L.; Burgert I.; Fratzl P. The Role of Wheat Awns in the Seed Dispersal Unit. Science 2007, 316 (5826), 884–886. 10.1126/science.1140097. 17495170

[ref2] Park Y.; Chen X. Water-responsive materials for sustainable energy applications. Journal of Materials Chemistry A 2020, 8 (31), 15227–15244. 10.1039/D0TA02896G.

[ref3] Chen X.; Mahadevan L.; Driks A.; Sahin O. Bacillus spores as building blocks for stimuli-responsive materials and nanogenerators. Nat. Nanotechnol. 2014, 9 (2), 137–141. 10.1038/nnano.2013.290. 24463362

[ref4] Chen X.; Goodnight D.; Gao Z.; Cavusoglu A. H.; Sabharwal N.; DeLay M.; Driks A.; Sahin O. Scaling up nanoscale water-driven energy conversion into evaporation-driven engines and generators. Nat. Commun. 2015, 6, 7346 10.1038/ncomms8346. 26079632 PMC4490384

[ref5] Quan H.; Kisailus D.; Meyers M. A. Hydration-induced reversible deformation of biological materials. Nature Reviews Materials 2021, 6, 264–283. 10.1038/s41578-020-00251-2.

[ref6] Armon S.; Efrati E.; Kupferman R.; Sharon E. Geometry and Mechanics in the Opening of Chiral Seed Pods. Science 2011, 333 (6050), 1726–1730. 10.1126/science.1203874. 21940888

[ref7] Park Y.; Jung Y.; Li T.-D.; Lao J.; Tu R. S.; Chen X. β-Sheet Nanocrystals Dictate Water Responsiveness of Bombyx Mori Silk. Macromol. Rapid Commun. 2020, 41 (7), 1900612 10.1002/marc.201900612. 32125047

[ref8] Jung Y.; Sharifi Golru S.; Li T.-D.; Biddinger E. J.; Tu R. S.; Chen X. Tuning water-responsiveness with Bombyx mori silk–silica nanoparticle composites. Soft Matter 2021, 17 (34), 7817–7821. 10.1039/D1SM00794G. 34612350

[ref9] Rabotyagova O. S.; Cebe P.; Kaplan D. L. Protein-Based Block Copolymers. Biomacromolecules 2011, 12 (2), 269–289. 10.1021/bm100928x. 21235251 PMC3071546

[ref10] Wang Y.; Katyal P.; Montclare J. K. Protein-Engineered Functional Materials. Adv. Healthcare Mater. 2019, 8 (11), 1970047 10.1002/adhm.201801374. PMC6703858 30938924

[ref11] Dai M.; Haghpanah J.; Singh N.; Roth E. W.; Liang A.; Tu R. S.; Montclare J. K. Artificial Protein Block Polymer Libraries Bearing Two SADs: Effects of Elastin Domain Repeats. Biomacromolecules 2011, 12 (12), 4240–4246. 10.1021/bm201083d. 22026464

[ref12] Haghpanah J. S.; Yuvienco C.; Civay D. E.; Barra H.; Baker P. J.; Khapli S.; Voloshchuk N.; Gunasekar S. K.; Muthukumar M.; Montclare J. K. Artificial Protein Block Copolymers Blocks Comprising Two Distinct Self-Assembling Domains. ChemBioChem. 2009, 10 (17), 2733–2735. 10.1002/cbic.200900539. 19806626

[ref13] Gunasekar S. K.; Asnani M.; Limbad C.; Haghpanah J. S.; Hom W.; Barra H.; Nanda S.; Lu M.; Montclare J. K. N-Terminal Aliphatic Residues Dictate the Structure, Stability, Assembly, and Small Molecule Binding of the Coiled-Coil Region of Cartilage Oligomeric Matrix Protein. Biochemistry 2009, 48 (36), 8559–8567. 10.1021/bi900534r. 19681593

[ref14] Olsen A. J.; Katyal P.; Haghpanah J. S.; Kubilius M. B.; Li R.; Schnabel N. L.; O’Neill S. C.; Wang Y.; Dai M.; Singh N.; Tu R. S.; Montclare J. K. Protein Engineered Triblock Polymers Comprised of Two SADs: Enhanced Mechanical Properties and Binding Abilities. Biomacromolecues 2018, 19 (5), 1552–1561. 10.1021/acs.biomac.7b01259. 29544048

[ref15] Sharma N.; Furter R.; Kast P.; Tirrell D. A. Efficient introduction of aryl bromide functionality into proteins in vivo. FEBS Lett. 2000, 467 (1), 37–40. 10.1016/S0014-5793(00)01120-0. 10664452

[ref16] Studier F. W. Protein production by auto-induction in high density shaking cultures. Protein Expr Purif 2005, 41 (1), 207–234. 10.1016/j.pep.2005.01.016. 15915565

[ref17] Savitzky A.; Golay M. J. E. Smoothing and Differentiation of Data by Simplified Least Squares Procedures. Anal. Chem. 1964, 36 (8), 1627–1639. 10.1021/ac60214a047.

[ref18] Micsonai A.; Wien F.; Bulyáki É.; Kun J.; Moussong É.; Lee Y. H.; Goto Y.; Réfrégiers M.; Kardos J. BeStSel: a web server for accurate protein secondary structure prediction and fold recognition from the circular dichroism spectra. Nucleic Acids Res. 2018, 46 (W1), W315–w322. 10.1093/nar/gky497. 29893907 PMC6031044

[ref19] Jackson M.; Mantsch H. H. The use and misuse of FTIR spectroscopy in the determination of protein structure. Crit Rev. Biochem Mol. Biol. 1995, 30 (2), 95–120. 10.3109/10409239509085140. 7656562

[ref20] More H. T.; Zhang K. S.; Srivastava N.; Frezzo J. A.; Montclare J. K. Influence of fluorination on protein-engineered coiled-coil fibers. Biomacromolecules 2015, 16 (4), 1210–1217. 10.1021/bm5019062. 25794312

[ref21] Agnarsson I.; Dhinojwala A.; Sahni V.; Blackledge T. A. Spider silk as a novel high performance biomimetic muscle driven by humidity. J. Exp Biol. 2009, 212 (13), 1990–1994. 10.1242/jeb.028282. 19525423

[ref22] Hill L. K.; Meleties M.; Katyal P.; Xie X.; Delgado-Fukushima E.; Jihad T.; Liu C.-F.; O’Neill S.; Tu R. S.; Renfrew P. D.; et al. Thermoresponsive Protein-Engineered Coiled-Coil Hydrogel for Sustained Small Molecule Release. Biomacromolecules 2019, 20 (9), 3340–3351. 10.1021/acs.biomac.9b00107. 31356057

