# Supplementary material for: Structure-Dependent Water Responsiveness of Protein Block Copolymers
Source: ACS Appl Bio Mater. 2024 May 15;7(6):3714–20. doi: 10.1021/acsabm.4c00045 (PMC11190970; doi:10.1021/acsabm.4c00045)
Supplement: Supplementary file 1 — mt4c00045_si_001.pdf [file mt4c00045_si_001.pdf]

Supporting Information

Structure-Dependent Water Responsiveness of Protein Block Copolymers

*Jacob Kronenberg[1, Δ], Yeojin Jung[2,3, Δ], Jason Chen[1], Maria Jinu Kulapurathazhe[1], Dustin Britton[1], Seungri Kim[2,3], Xi Chen[2,3,4, *], Raymond S Tu[3,*], Jin Kim Montclare[1,5,6,7,8,*]*

1 New York University Tandon School of Engineering, Department of Chemical and Biomolecular Engineering, Brooklyn, New York, 11201, USA

2 City University of New York, Advanced Science Research Center (ASRC) at the Graduate Center, New York, New York, 10031, USA

3 City College of New York, Department of Chemical Engineering, New York, New York, 10031, USA

4 City University of New York, PhD Programs in Chemistry and Physics at the Graduate Center, New York, New York, 10016, USA

5 New York University, Department of Chemistry, New York, New York, 10031, USA

6 New York University College of Dentistry, Department of Biomaterials, New York, New York, 10010, USA

7 New York University Grossman School of Medicine, Department of Radiology, New York, New York, 10016, USA

8 New York University Tandon School of Engineering, Department of Biomedical Engineering, Brooklyn, New York, 11203, USA

Δ J.B.K. and Y.J. contributed equally.

* Corresponding authors

Email: montclare@nyu.edu; tu@ccny.cuny.edu; xchen@gc.cuny.edu

Protein Biosynthesis. Representative SDS-PAGE gels revealed a band around 27 kDa in the post-induction samples but not in the pre-induction samples, meaning that CEC and CEC_{L44A} were successfully expressed (**Fig. S1A**). SDS-PAGE gels of metal affinity column elutions showed bands around 27 kDa and 54 kDa corresponding to the CEC or CEC_{L44A} monomer and dimer respectively (**Fig. S1B**). MALDI-ToF-MS agreed with gel electrophoresis and confirmed the identification of the two bands as pure protein of interest (**Fig. 1**).

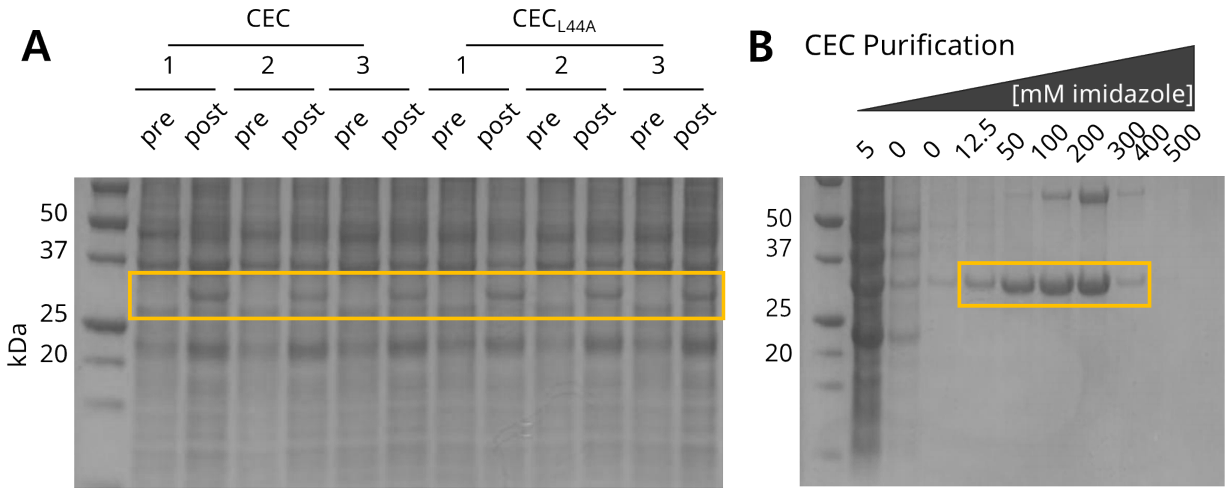

Figure S1. A. a representative SDS-PAGE gel containing bands corresponding to CEC and CEC_{L44A} in the post-induction samples confirming expression, **B.** a representative SDS-PAGE gel with bands at 27 kDa and 54 kDa corresponding to the CEC monomer and dimer.

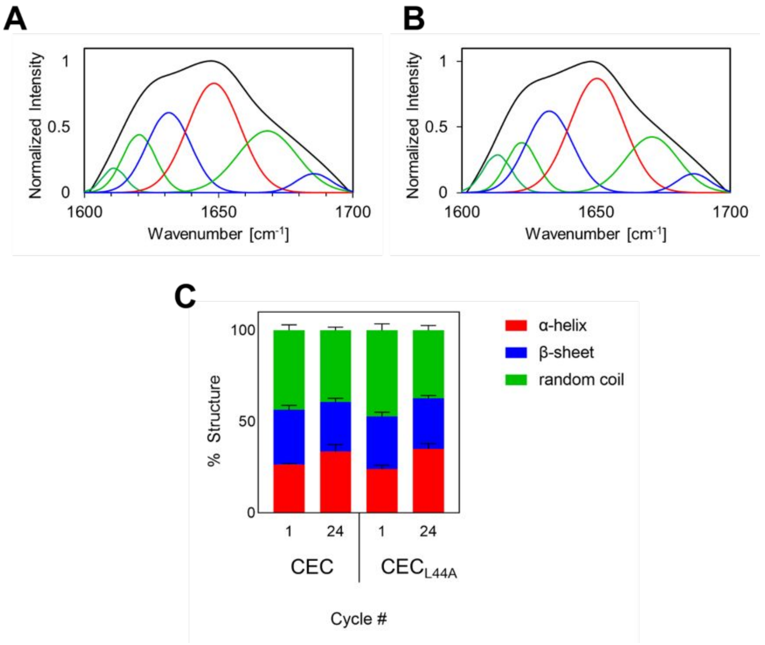

Figure S2. Representative ATR-FTIR spectra of **A)** CEC and **B)** CEC_{L44A} films after 24 RH cycles. **C)** Percent contributions to secondary structure of CEC and CEC_{L44A} after 24 RH cycles. Error bars represent standard deviations calculated from 3 independent measurements.

Secondary Structure Percent. Secondary structure fractions were determined by analysis with BeStSel for CD and deconvolution in PeakFit for ATR-FTIR.

	**CD**		**ATR-FTIR (before cycling)**		**ATR-FTIR (after cycling)**	
**Percent (%)**	CEC	CEC_{L44A}	CEC	CEC_{L44A}	CEC	CEC_{L44A}
**α-helix**	39.7 ± 3.2	9.1 ± 1.8	26.5 ± 0.6	24.0 ± 2.3	33.7 ± 3.7	35.1 ± 2.9

**β-sheet**	35.6 ± 4.0	45.9 ± 2.4	30.0 ± 2.4	28.8 ± 2.3	27.0 ± 2.0	27.6 ± 1.6
**random coil**	24.7 ± 0.6	45.0 ± 1.1	43.5 ± 3.0	47.2 ± 3.5	39.3 ± 1.7	37.3 ± 2.6

Table S1. Secondary structure percent of CEC and CEC_{L44A} as calculated from CD and FTIR spectroscopy showing that CEC has a higher α-helical content than CEC_{L44A} under both wet (CD), dry (ATR-FTIR) conditions before and after RH cycling.

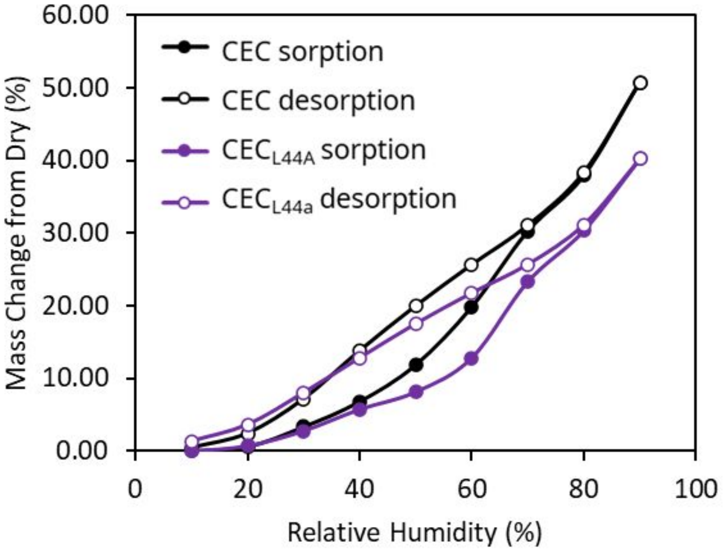

Figure S3. DVS water sorption isotherms showing that lyophilized CEC powder takes up slightly more water than lyophilized CEC_{L44A} powder and that both show minimal hysteresis during desorption.

Surface Characterization. Scanning electron micrographs of CEC and CEC_{L44A} were taken at 10K and 30K magnification. Micrographs reveal a continuous surface on the protein strips (**Fig. S4)**. Both variants show nanoporous surfaces with pores evenly distributed across the strip, with

no immediately apparent differences in macrostructure among batches of strips or between strips made with the two variants. Image analysis showed that the pore sizes did not differ, with CEC having a pore size of 31.6 ± 9.5 nm and CEC_{L44A} having a pore size of 32.0 ± 9.8 nm. Pore area made up 2.1 ± 0.3% of the surface of the CEC samples but 4.7 ± 1.2% of the surface of the CEC_{L44A} samples. This suggested that one reason for CEC_{L44A}'s stronger stimulus response could be due to improved transport of water into the protein active layer due to greater pore volume.

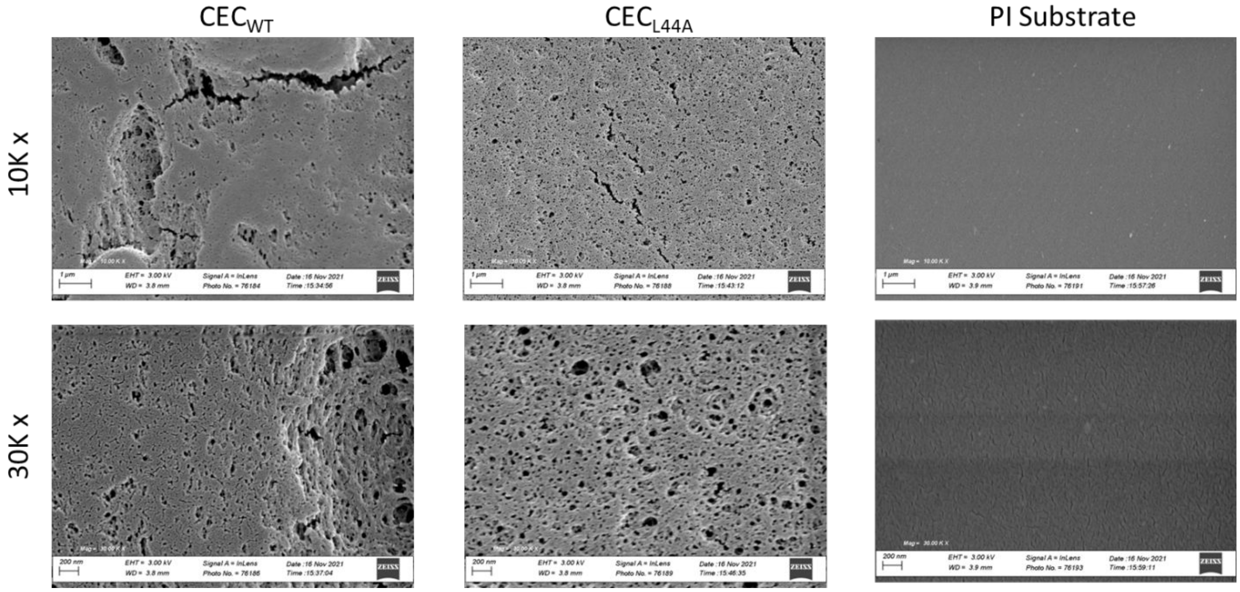

Figure S4. Representative scanning electron micrographs of the surfaces of CEC_{WT} and CEC_{L44A} thin layers cast onto polyimide strips as well as plain polyimide substrate strips, shown at 10K and 30K times magnification. Scale bar is 1 μm.

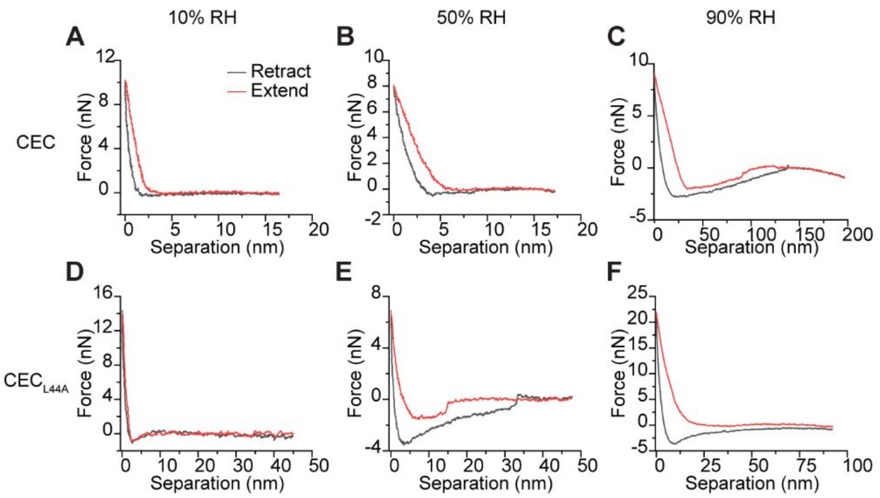

Figure S5. Representative force-separation curve for AFM Young's moduli of CEC (A-C) and CEC$_{L44A}$ (D-F) measurements at 10%, 50%, and 90% RH.
